# ‘Just another incentive scheme’: a qualitative interview study of a local pay-for-performance scheme for primary care

**DOI:** 10.1186/s12875-014-0168-7

**Published:** 2014-10-25

**Authors:** Julia Hackett, Liz Glidewell, Robert West, Paul Carder, Tim Doran, Robbie Foy

**Affiliations:** Leeds Institute of Health Sciences, University of Leeds, Charles Thackrah Building, 101 Clarendon Road, Leeds, UK; West and South Yorkshire and Bassetlaw Commissioning Support Unit, Douglas Mill, Bowling Old Lane, Bradford, UK; Department of Health Sciences, University of York, Rowntree Building, York, UK

**Keywords:** Primary health care, Pay-for-performance, Financial incentives, Social deprivation

## Abstract

**Background:**

A range of policy initiatives have addressed inequalities in healthcare and health outcomes. Local pay-for-performance schemes for primary care have been advocated as means of enhancing clinical ownership of the quality agenda and better targeting local need compared with national schemes such as the UK Quality and Outcomes Framework (QOF). We investigated whether professionals’ experience of a local scheme in one English National Health Service (NHS) former primary care trust (PCT) differed from that of the national QOF in relation to the goal of reducing inequalities.

**Methods:**

We conducted retrospective semi-structured interviews with primary care professionals implementing the scheme and those involved in its development. We purposively sampled practices with varying levels of population socio-economic deprivation and achievement. Interviews explored perceptions of the scheme and indicators, likely mechanisms of influence on practice, perceived benefits and harms, and how future schemes could be improved. We used a framework approach to analysis.

**Results:**

Thirty-eight professionals from 16 general practices and six professionals involved in developing local indicators participated. Our findings cover four themes: ownership, credibility of the indicators, influences on behaviour, and exacerbated tensions. We found little evidence that the scheme engendered any distinctive sense of ownership or experiences different from the national scheme. Although the indicators and their evidence base were seldom actively questioned, doubts were expressed about their focus on health promotion given that eventual benefits relied upon patient action and availability of local resources. Whilst practices serving more affluent populations reported status and patient benefit as motivators for participating in the scheme, those serving more deprived populations highlighted financial reward. The scheme exacerbated tensions between patient and professional consultation agendas, general practitioners benefitting directly from incentives and nurses who did much of the work, and practices serving more and less affluent populations which faced different challenges in achieving targets.

**Conclusions:**

The contentious nature of pay-for-performance was not necessarily reduced by local adaptation. Those developing future schemes should consider differential rewards and supportive resources for practices serving more deprived populations, and employing a wider range of levers to promote professional understanding and ownership of indicators.

## Background

Evidence is accumulating that the establishment in 2004 of the Quality and Outcomes Framework (QOF) as a pay-for-performance scheme for UK primary care has not fulfilled all hopes and expectations [1]. Not only is there a problematic evidence base [2], but its effects appear mixed [3] with persistent variations in the quality of primary care [4] and concerns that QOF may have undermined professionals’ intrinsic motivation, patient-centeredness, and continuity of care [3,5-9]. Professionals are reluctant to engage in quality improvement initiatives perceived as ineffective or even harmful [10], including pay-for-performance schemes misaligned with professional values [1,6,11-13]. The Darzi Review of quality improvement in the National Health Service (NHS) placed much emphasis on engaging professionals [14]. At a local level, active involvement of professionals is presumed essential in promoting ownership, providing that perceived benefits of change compensate for the effort required [15-17]. At face value, the establishment of pay-for-performance schemes with locally negotiated indicators offered advantages over the national scheme, as means of promoting clinical ownership by addressing local health priorities and enhancing the effects of incentives [18].

We evaluated a scheme in one former PCT which was particularly motivated by the need to address inequalities in healthcare provision and outcomes. The scheme ran over 2007–11 at a cost of £3 million, and targeted five health priorities: alcohol; learning disabilities; chlamydia; obesity; and osteoporosis (Table 1). The selection of priorities, indicators and payment thresholds were negotiated between the PCT and local health care providers, approved by the Local Medical Committee, and reviewed and refined over the lifetime of the scheme. Our accompanying paper provides more detailed information about the indicators [19]. We found that gaps in achievement between practices serving less and more deprived patients were modest during the first year of the scheme and closed over time for one and widened for one of the 16 indicators and possibly two other indicators. In addition, larger practices and those serving more affluent areas earned more income per patient than smaller practices and those serving more deprived areas.

**Table 1:** **Indicators for the local pay-for-performance scheme**

**Domain**	**Indicator**	**Description**	**Number of points**
Alcohol	A1	The practice can produce a register of patient aged 16 years and over with a record of the number of units of alcohol consumed on a weekly basis in the past 27 months	10
	A2	Patients who drink equal or greater than 14 units a week for females and 21 units a week for males in a 7 day cycle with a period of at least 2 days abstinence are offered a brief intervention	10
Chlamydia	C1	The practice can produce a register of patients aged 15 to 24 of both sexes	2
	C2	Patients between 15–24 years old who have been offered screening by their practice and have a recorded test result	£5 for every screen recorded
Learning Disabilities	LD1	The practice can produce a register of people over 18 with LD	£50 per registered patient
	LD2	The % of patients with LD with a review recorded in the preceding 15 months. Checks include accuracy of prescribed medication, physical health and co-ordination with secondary care	£50 for every health check completed
Weight Management	OB1	Production of a register of patients between 16–75 with a BMI equal of greater than 25 recorded in the last 5 years	3
	OB2	Production of a register of patients between 16–75 with a BMI equal of greater than 25 recorded in the last 15 months	7
	OB3	Patients with a BMI equal or greater than 25 receive appropriate intervention in the past 15 months	20
Osteoporosis	OST1	Production of a register of female patients aged 65–74 with a fracture in the previous 15 months	2
	OST2	Female patients 65–74 that have had a fracture are referred for a BMD scan	4
	OST3	The practice can produce a register of male and female patients aged 16–74 years who have received at least one repeat prescription for oral prednisolone in the previous 6 months	2
	OST4	The % of patients on register (OST 3) who have a record of a DXA scan being performed at any time or a referral for a DXA scan in the previous 15 months	5
	OST5	The percentage of patients on register (OST 4) who have a record of a DXA scan being performed at any time, or a referral for a DXA scan in the previous 15 months, or have been assessed for osteoporosis risk	2
	OST6	The practice can produce a register of male and female patients aged 75 years and over who have had a fragility fracture of the vertebrae, hip, wrist, or humerus since their 75th Birthday	2
	OST7	The percentage of male and female patients aged 75 years and over who have had a fragility fracture of the vertebrae, hip, wrist, or humerus since their 75th Birthday, who have been assessed and treated for Osteoporosis risk ever	5

These mixed findings somewhat contrasted with longitudinal analyses of the national QOF which indicated that initial gaps in achievement between practices in deprived and affluent areas these closed over time [20]. It was disappointing that a local initiative intended to overcome the disadvantages of the national scheme did not reduce inequalities as intended.

We undertook a qualitative study, in parallel to our above quantitative analysis, to explore primary care professionals’ experience of the local QOF, including perceptions of the scheme and indicators, likely mechanisms of influence on practice, and perceived benefits and harms. We investigated whether professionals’ experience of the local QOF did differ from that of the national QOF in relation to the goal of reducing inequalities.

## Methods

### Design and setting

We undertook a retrospective semi-structured interview study within NHS Bradford and Airedale, of its local pay-for-performance scheme.

### Participants

We initially invited managers from all 83 practices to nominate themselves and other practice staff to participate in interviews. We then purposively selected practices according to practice population socio-economic profiles (deprived or not) and local QOF achievement (high or low achievement). We then used snowballing to further recruit participants through asking those interviewed to nominate additional practices or participants. We also invited six PCT and practice professionals involved in developing the scheme.

### Data collection and analysis

Following consent, a social scientist researcher (JH) conducted face-to-face interviews at venues of participants’ choice (usually at work) over August 2011 to June 2012. We reimbursed participants for their time and advised them that responses would be treated confidentially. Interviews explored whether perceptions of the indicators, mechanisms by which it influenced practice, benefits and harms, and how future iterations of such schemes could be improved (Topic guide, Table 2).

**Table 2:** **Topic guide**

**Section**	**Types of questions/prompts**
**Background**	What is your professional background?
	How many years have you been qualified?
	How many sessions do you work in a usual week?
	How would you describe your role in the practice?
**General**	What has your involvement been in developing the local QOF?
	What has your involvement been in implementing the local QOF within your practice?
**Your opinions**	
Appropriateness of incentivised targets	Robustness/credibility of evidence base
	Costs
Relevance	Clinical benefit
	Local population needs
Fairness of indicators	Distribution of workload
	Scope for gaming
	Implications for tackling inequalities
Acceptability of targets	Compare to national targets
**How does the local scheme work?**	
How does the scheme influence what you do?	Ownership of change/engagement
	Motivation (intrinsic and extrinsic)
	Social comparison, performance management and surveillance
	Organisational means employed to achieve targets
**Consequences**	Effect on practice staff and consultations
	- Benefits and unintended consequences
	Effect on patients and patient care
	- Benefits and unintended consequences
	Change required to achieve targets
	Are you still maintaining these targets even though the scheme has ended?
**How could local QOF be modified and/or improved?**	How it should be introduced
	How implemented on a day to day basis in the practice
	Local versus national benefits and harms?
**Anything else that you would like to add?**	

All interviews were recorded and transcribed verbatim. Transcripts were anonymised and checked for accuracy. We used NVivo 8 to manage interview data and a thematic framework approach to analysis [21]. Five transcripts were double coded by (JH, LG and RF) and a coding schedule was developed (Table 3). JH coded the remainder of the transcripts. Data were initially coded deductively to areas pre-specified in the topic guide; further codes emerged from the data inductively. Codes were grouped to form overarching themes which were iteratively refined over the course of analysis. Recruitment and interviews continued until no new codes had emerged. We compared and contrasted accounts from high and low deprivation and high and low achieving practices, and sought discrepant accounts.

**Table 3:** **Coding schedule**

**Deductive coding to areas taken from literature**	**Inductive codes emerging from interviews**	**Iterative refining of deductive and inductive codes and themes**		**Final themes**
**Influences on behaviour:**		**Motivation:**	**Practitioner motivation:**	**Influences on behaviour**
Ownership of change	Support among practices	Patient benefit	Financial reward	
Motivation (intrinsic and extrinsic)	Financial reward		Patient benefit	
			Competition with other practices	
Social comparison				
Organisational means				
**Relevance:**		**Opinions:**	**Attitudes towards the scheme:**	
Clinical benefit	Clinical value	Don’t agree with localisation	Role of general practice	
Local population needs	Credibility	Lack of knowledge/interest in evidence	Acceptance/rejection of an externally defined way of working	
	Prevalence		Faith in the evidence	
**Fairness:**				
Distribution of workload	Uneven workload			
Scope for gaming	Minimal change			
Implications for tackling inequalities	The bigger picture			
	Failed to address inequalities			
	Adjusting role of general practice			
**Appropriateness of incentivised targets:**		**Credibility:**		**Credibility of the locally negotiated indicators**
Robustness of evidence base	Conflict with professional identity	Other guidelines		
Costs	Conflict among practice staff	Clinical value		
	Conflict with patient benefit	Conflict with/supported by prevalence in population		
	Funding improves credibility			
**Acceptability:**		**Effect on professionals:**	**Effects of implementing a local scheme:**	**Exacerbating tensions**
Compare to national QOF	Just another income stream	Created an uneven workload	Allowed local issues to be addressed	
	Conflicting credibility with NQOF		Caused inequalities	
**Consequences:**		**Effect on patients:**	**Consultation consequences**	
Effect on practice staff	Adapt consultations	Standardised care	Target became routine practice	
		**Effect on consultations:**		
		Adapt templates as aids		
Effect on patients and patient care	Impact on patient experience	Embedded behaviour		
	Time pressure	Required minimal change		
	Conflicting agendas			
	Distracting in consultations			
	Embedded behaviour			
	Standardised care			
**Recommendations:**		**Recommendations:**	**Experience of engagement**	**Ownership**
How it should be introduced	Evolving assessment process	LoQOF champion	Highlight available external support for data extraction and management	
Local versus national benefits and harms	Extension of NQOF	Patient involvement	Familiarisation period before data collection	
	Conflict with NQOF	Bottom up approach		
	Bottom up approach	Based at cluster level		
	Setup time	Outside support		
		Protected learning time for *all* staff		
		Data support		

### Ethical review

The study was approved by National Research Ethics Service East Midlands- Nottingham 2 Committee (11/EM/0184).

## Results

We interviewed 44 professionals involved in developing or implementing the local scheme. Primary care staff from 16 practices participated in the interviews, eight of these practices having been identified through snowballing. Eight practices served relatively socio-economically deprived populations and 12 had relatively high local QOF achievement (Table 4). Of the 38 practice staff interviewed, there were 15 practice managers, 10 GP partners, two salaried GPs, and 11 practice nurses. The six additional participants who had been involved in developing the scheme comprised four PCT managers, one salaried GP, and one practice nurse. Thirty-three participants were female and 24 worked full-time. Median interview length was 44 minutes (range 18 to 88 minutes).

**Table 4:** Spread of practices and practice staff across performance and deprivation*

**QOF score**	**Deprivation level**	
	**Deprived**	**Affluent**
**High**	**5 practices**	**7 practices**
	- GP Partner (3)	- Practice Manager (8)
	- Practice Nurse (2)	- GP Partner (5)
	- Practice Manager (5)	- Practice nurse (8)
		- Salaried GP (2)
**Low**	**3 practices**	**1 practice**
	- Practice Manager (1)	- Practice nurse (1)
	- GP Partner (2)	- Practice Manager (1)

*In addition, there were six other people interviewed who were involved with the development of the local scheme: four PCT members, one salaried GP, and one practice nurse.

We report our findings in four overarching themes: credibility of the locally negotiated indicators; ownership; influences on behaviour; and exacerbated tensions. Where evident, we compare and contrast findings according to participants’ practice population socioeconomic status and achievement, and involvement in scheme development.

### Credibility of the indicators

The local scheme developers had sought to target locally relevant and, largely, public health issues absent from the national QOF. Professionals perceived the limited evidence base underpinning such indicators as less of an issue than practical considerations around their implementation. Hence, the evidence base was often taken at face value, especially by practice nurses:

‘We appreciate that it is evidence based, obviously we wouldn’t be been asked to do anything that wasn’t.’ (P11, practice nurse, high performer, affluent area).

‘I don’t know if I was told about the evidence, we should say, “What’s the evidence behind this?” but we’re too busy.’ (P37, practice nurse, high performer, deprived area).

Professionals appeared more preoccupied by their lack of control in achieving indicator targets, especially if dependent upon patient cooperation:

‘I can see why the alcohol and obesity were thought of as important, I get the clinical reason but I’m not sure that it worked in the real world. People thought we’d get them in and we’d do this, but the fact is that they don’t come in and you don’t capture them and so it doesn’t work.’ (P19, practice manager, high performer, affluent area).

Limited availability of appropriate, supportive resources needed to address such problems further undermined confidence in these targets.

‘We’ve got a smoking cessation advisor within the practice, but there isn’t something with alcohol, and you wouldn’t refer to the alcohol and drugs services unless someone’s quite bad.’ (P12, salaried GP, high performer, affluent area).

There was a range of opinion about relevance to local need, with the indicators being seen as more salient to relatively deprived populations.

“It was certainly developed based on looking at measureable things that were relevant to our population.’ (P36, GP partner, low performer, deprived area).

In contrast, professionals from practices in affluent areas questioned the value of certain indicators to their population.

‘The alcohol one for example for us is almost a bit of a waste of time, because our patients don’t fall into that category.’ (P11, practice nurse, high performer, affluent area).

### Ownership

No clear sense emerged that the local pay-for-performance scheme was particularly distinctive and offered anything over and above the existing national QOF. This was partly because the scheme actually addressed national priorities.

‘We know too many people are overweight so in that sense it was targeted at areas where we had a particular problem…I’m not aware that we had a specific problem with osteoporosis in Bradford, likewise with learning disabilities, I don’t think we’ve got any more of an issue than other areas. There may have been other Bradford specific issues that we could have included which we didn’t…I think most GPs probably viewed it as just another incentive scheme, and didn’t really think of it as bespoke.’ (P6, scheme developer).

Ultimately then, practices tended not to differentiate between national and local schemes, especially high performers.

‘It makes me feel no different, it’s just all part of my job, whether it’s a local thing or national, it makes no difference.’ (P19, practice manager, high performer, affluent area).

One practice manager in a low performing practice went further in stating that the national scheme was more important.

‘We were always aware it (the local scheme) was there but we didn’t feel it was as important as the (national) QOF.’ (P39, practice manager, low performer, affluent area).

Participants implicitly defined ‘local’ in different ways, including at the practice, cluster of practices, and PCT levels.

‘I think smaller cluster groups, because generally you’ll have an area such as ourselves here with about twelve surgeries where we’ve all got similar problems, so I think it would have helped if practices were grouped rather than it being a generic local QOF.’ (P14, practice manager, low performer, deprived area).

There was a further suggestion that ‘buy-in’ might be greater if the identification of at least a limited number of priorities were delegated to practice level.

‘From the start you’d be making them own it because you’d be saying “right, here’s a bit of money, you tell us how you want to spend it as a practice to improve quality of your patients”, so you’ve got the ownership immediately because they’ve come up with the marker.’ (P10, practice manager, high performer, affluent area).

Some participants expressed views that initial dissemination was insufficient and a familiarisation period would have helped embed targeted behaviours.

‘If we’d been told a bit more we might have been more engaged.’ (P23, practice nurse, high performer, affluent area).

‘If we had time to play about with it and start to monitor our own performance that would be really useful.’ (P10, practice manager, high performer, affluent area).

### Influences on behaviour

The scheme seemed to influence adherence to the targets primarily through motivational means, supported by other mechanisms. Motivations were extrinsically and intrinsically driven.

Professionals from practices serving both affluent and deprived populations felt the scheme legitimised their intrinsic motivation to improve patient outcomes.

‘It’s a massive motivation to know that the patients out there are getting the care that they need.’ (P39, practice manager, low performer, affluent area).

Others, particularly practices serving more deprived populations, appeared to be directly amenable to financial reward as an extrinsic driver.

‘We’re so hard up at the moment, so desperate for income wherever we can get it, you can’t afford to pass up a chance of income, so that’s probably as much a driver…even if we didn’t necessarily buy in completely to the clinical benefit, it was worth doing to try and earn the money because we needed to.’ (P33, practice manager, high performer, deprived area).

However, there were concerns that financial rewards from the scheme may not have been worth the effort involved in achieving targets and that the scheme did not directly target most of the people actually doing this additional work.

‘Yes it’s more money for the practice but the majority of people in general practice are paid by the practice and they just see it’s more work for them to do, certainly our practice staff used to think of it [Local QOF] as a huge amount of work’ (P4, scheme developer).

For practice managers and GPs in affluent high-performing practices, competition and implicit threats to status also emerged as motivators.

‘It does feel a bit like a competition with other surgeries, I don’t know how others feel but I wouldn’t like to come last in our locality.’ (P19, practice manager, high performer, affluent area).

There were three other ways in which the scheme appeared to influence clinical behaviour. Firstly, several high-performing practices and one low-performer had adapted templates provided by the PCT to support processes of care and recording in consultations. Practitioners from these practices considered that such prompts had been helpful.

‘Before the patients come in you know that you have to do these things, so it is a motivation. If the reminder didn’t come up, you wouldn’t remember to do those things.’ (P22, GP partner, low performer, deprived area).

Secondly, some health professionals and developers of the scheme felt that it promoted standardised care and believed that adherence to the indicators had become routine practice. Consultation templates supported this setting of new norms within clinical routines.

‘Once we start doing something, it does change your practice and you carry on. The learning disabilities, because we saw the value of it we’ve kept the template, we’re still doing the checks, so I think because we put in all that initial time and resource, actually then each year it will get less, so we’re happy to carry that on. I think where we’ve seen that there’s clinical benefit, once you start doing it, it becomes habit.’ (P27, salaried GP, high performer, affluent area).

Thirdly, the social influence of having a member of practice staff as the champion for the scheme promoted engagement.

‘It’s having someone that’s responsible for it, it’s their baby, they’ve got an interest in it, and they will drive it through. That’s what you need if you want to achieve with these things you need a champion, someone who will champion it for you.’ (P33, practice manager, high performer, affluent area).

### Exacerbated tensions

The scheme exacerbated tensions at three levels: between patients and professionals within consultations; between doctors and nurses within practices; and between affluent and deprived population practices within the PCT.

Perceived pressure to focus on targets and ‘box ticking’ during consultations both undermined professionalism and alienated patients.

‘A lot of patients know I’m ticking a box and they shouldn’t feel like that, a patient shouldn’t have to come to a surgery and then I just say, “Oh can I ask you this”, “Oh yeah you’re just ticking, ticking that box.” They shouldn’t feel like that.’ (P40, practice nurse, low performer, affluent area).

This generated conflict between GP and patient agendas, which many also recognised as a consequence of the national QOF.

‘It distracts from the consultation and it can leave you know feeling a bit confused and perhaps as though that, the thing that the patient regards as the problem hasn’t been addressed properly.’ (P6, scheme developer).

There were also concerns about adding more and more into consultations:

‘The consensus among a lot of the GP’s was that it moved away from being patient centred to doctor centred consultations in that we never actually got round to why the patient really had come to see us if we spent so much time on QOF. There was a lot of discussion around running out of time and then running over, and the impact that that had on the patient, the practice and then personally. (P29, GP partner, high performer, affluent area).

The scheme augmented perceptions of unfair distributions of workloads and remuneration within practices, particularly between nursing and medical staff. Some nurses were keen to emphasize that they did not think that they should receive additional money for doing their job.

‘We’re paid money to do that anyway, why is it that there’s extra money given when you’re given a wage to do it anyway? I don’t know why a carrot should be dangled to a health professional, personally I find it immoral.’ (P37, practice nurse, high performer, deprived area).

However, several nurses were openly critical of the fact that whilst they did most of the work, it was the GPs who benefitted financially.

‘I think we feel that we do a lot of work towards the QOF and we probably feel as though we ought to recompensed, if we had a bonus that was specifically because we knew that we’d hit QOF targets. I think people feel well why should only certain parts of the team get it when everybody’s worked as hard towards it?’ (P11, practice nurse, high performer, affluent area).

Amongst practices serving relatively affluent and deprived populations, there was an opinion that the scheme risked widening inequalities between ‘us and them’ if universally applied, as opposed to focusing on practices and populations with most scope for improvement:

‘You’ll always get this top lot that will sign up to it all, always do it, know how to do it, cause they’re whizzes. But you’ve always got the laggards at the bottom. They’re the ones that really need to be doing the local QOF. It really should have been targeted at those practices first.’ (P13, practice manager, high performer, affluent area).

## Discussion

Contrary to aspirations, this local pay-for-performance scheme did not engender any distinctive sense of ownership nor avoid any of the conflicts associated with the national scheme. The indicators were seen as reflecting national rather than specifically locally-owned priorities; subsequent to the initiation of this scheme, three out of the five health priorities had been included in the national QOF. Although the indicators and their evidence base were seldom actively questioned, doubts were expressed about their focus on health promotion given that eventual benefits relied upon patient action and the availability of local resources (e.g. for alcohol or weight problems).

Whilst practices serving more affluent populations focused on status and patient benefit as motivators for participating in the scheme, those serving more deprived populations also highlighted financial reward. However, the scheme appeared to influence behaviour through a range of mechanisms beyond extrinsic reward such as standardisation of patient care, practice champions and computerised prompts. Unintended consequences included the exacerbation of tensions at three levels: between patient and professional consultation agendas; between GPs seen as benefitting directly from incentives and nurses who did much of the work; and between practices serving more affluent populations where targets might be easier to achieve and those serving more deprived populations.

There has been relatively little evaluation of local pay-for-performance schemes, which are likely to continue emerging in various forms [18]. We identified similar themes to qualitative studies of the national QOF scheme, including the credibility of incentivised targets, tensions within consultations, changing professional identity and roles, and inequities in the workload and remuneration balance among practice staff [5-9,22-25]. These suggest that the local scheme was not viewed or experienced differently by targeted professionals and, taken with our findings suggesting sparse ownership, casts doubt upon the notion that such a scheme achieved greater professional ‘buy-in.’ Our findings are therefore consistent with an evaluation by Kristensen et al. of a national pay-for-performance initiative which centred on locally negotiated indicators [26]. This also found a gap between the policy intention of creating locally-owned indicators and actual experience of the initiative. Interventions aiming to improve the quality of care are often conceived and implemented based on a hopeful set of assumptions about professional behaviour and contexts [27]. Like others, we found that this scheme appeared to operate in a number of ways, beyond the direct influence of financial incentives [6,22,28]. Hence, the range of explicit and implicit behaviour change techniques associated with pay-for-performance schemes, such as social influence and competition, underline the need to conceptualise and evaluate them as complex interventions [29-31]. Again, the notion of local ownership did not emerge as a strong additional driver for change in our evaluation.

Our study limitations included the experiences of an intervention from the one former PCT, the characteristics of participating practices, study participants and timing, and the risk of social desirability bias. First, this study took place in one geographical area and studied one local pay-for-performance scheme, thereby limiting generalizability to other areas and schemes. Second, although we sought a range of practice characteristics for our sample, we found that our participants under-represented poorer performing practices. This could have affected the balance of views and experiences, potentially towards an emphasis on positive experiences. However, we encountered sceptical beliefs across the range of participants, even amongst scheme developers. Third, we examined perspectives of both those targeted by the scheme and its developers, and encountered little divergence of views. We might have identified more differences had we been able to capture the developers’ ideas and expectations during the planning phase of the scheme. We were unable to identify further information on how the indicators were ‘evidenced,’ which may have influenced perceived credibility. Fourth, we were aware that professionals interviewed might tend to express socially desirable opinions or behaviours. This could have steered responses either way – towards being seen either to favour the scheme or critical of the PCT. We emphasized the anonymity and confidentiality of study participation, and the interviews did not aim to judge professional performance.

Potential indicators require testing for key attributes such as acceptability and feasibility before they can be rolled out nationally [32]. Glasziou and colleagues proposed nine criteria to help judge whether incentive schemes are likely to do more good than harm [33]. Three of these seem particularly relevant viewed through the lens of health professionals targeted by a local scheme: whether the desired clinical action improves patient outcomes; whether benefits clearly outweigh any unintended harmful effects, and at an acceptable cost; and whether systems and structures needed for change are in place.

The Bradford and Airedale scheme’s focus on public health priorities – in contrast to the national QOF which largely focuses on clinical monitoring and treatment – illustrates some of the challenges inherent in fulfilling these criteria. Some health professionals believed that the local preventive targets could be cost-effective in the long-term. Others expressed uncertainty about their ‘real world’ effects, reflecting wider doubts about their roles and competencies in promoting health [34-36] and concerns that attainment depended upon patient adherence or supporting resources in the wider community. Any perceived benefits may have been outweighed by unintended knock-on effects on a range of professional and patient relationships [25].

“Localism” is regularly recycled as a theme in NHS policy-making [37]. In order to increase clinical autonomy and therefore have maximal impact upon patient care, there are continuing calls for greater professional involvement in developing pay-for-performance indicators [38]. This is order to increase professional buy-in with such schemes and ensure that indicators are developed from within and not imposed from the outside [26]. Yet it is difficult to get beyond such rhetoric in practice, particularly in generating and implementing performance targets which are perceived as locally relevant and owned. Professionals tend to voice opinions about the need for more involvement in developing targets and their dissemination. In reality, there are only so many consultations, working groups or educational events that they can actually participate in. Furthermore, local groups are unlikely to have access to similar levels of resources, such as those possessed by the National Institute for Care Excellence, to derive robust, evidence-based indicators. There is a case for further efforts to ensure that the underlying goals of performance targets are communicated to targeted professionals and aligned with professional values, especially as a means of overcoming some of the passive acceptance we found [11,12,22]. There is a growing and increasingly robust evidence base on interventions to change professional practice for policy-makers and quality improvement leaders to draw upon [39].

Pay-for-performance itself has a problematic evidence base, with a Cochrane Review concluding there is “insufficient evidence to support or not support the use of financial incentives to improve the quality of primary health care” [2]. Given that one of the intentions of such schemes is often to reduce inequalities in health outcomes, any future local schemes may need to recognise the greater difficulties faced by practices serving more deprived populations [40]. As well as financial reward, suggested as a stronger motivator in such practices, the achievement of indicators may also depend upon resources already available within practices and the wider community. Persuasion about patient benefit and social comparison were also critical levers, or implicit co-interventions. Pay for performance represents an inherently complex intervention with variable effects according to context, the nature of the behaviours targeted, and co-interventions, all of which need to be taken into account in planning and evaluating such schemes [28,41].

Policy-makers should not under-estimate the difficulties faced in promoting ownership of local pay-for-performance schemes. Incentives alone are often insufficient to bring about change; significant progress is likely to depend upon multi-level approaches which launch and coordinate action across all levels of healthcare systems (individual, team, organisational and wider system) [42]. These approaches should draw upon evidence-based interventions to improve practice [39], tailored to identified barriers to change. The costs of efforts to promote engagement with local pay-for-performance schemes need to be considered against realistic appraisals of their likely effects and alternative strategies.

## Conclusion

We found little difference in the experience of a local pay-for-performance scheme compared to a national scheme. Together, with the limited evidence of professional ownership, it is hard to argue that it offered distinct advantages over and above the existing national QOF scheme. Future developments of similar schemes should study the impact of differential rewards for practices serving more and less deprived populations, and consider a wider range of levers to promote professional understanding and ownership of indicators.

## Abbreviations

PCT: Primary care trust
QOF: Quality and outcomes framework
GP: General Practitioner
NHS: National Health Service
